# Self-reported exposure to pesticides in residential settings and risk of breast cancer: a case-control study

**DOI:** 10.1186/1476-069X-9-30

**Published:** 2010-06-25

**Authors:** Umar Farooq, Monika Joshi, Vinod Nookala, Pramil Cheriyath, Daniel Fischman, Nora J Graber, Steven D Stellman, Joshua Muscat

**Affiliations:** 1 Department of Internal Medicine, Harrisburg Hospital, Pinnacle Health Hospitals, Harrisburg, PA 17104, USA; 2 Department of Public Health Sciences, Penn State College of Medicine, Hershey, PA 17033, USA; 3 Department of Epidemiology, Mailman School of Public Health, Columbia University, New York City, NY 10032, USA

## Abstract

**Background:**

Pesticides are widely used in households to control insects and weeds. Several studies, over the past decades, have examined the possible relationship of serum concentration of organochlorine pesticides and the development of breast cancer. However, little data exists regarding an association between self-reported, residential exposure to pesticides and breast cancer risk. We, therefore, present a case-control study examining self-reported exposure to household pesticides with regard to associated risk of breast cancer.

**Methods:**

This study was conducted in the area in and around New York City, NY and included 1205 patients (447 cases and 758 controls). Cases were defined as women with newly diagnosed breast cancer or carcinoma in-situ, while controls included women with benign breast diseases or those undergoing non-breast related surgery. All patients were asked a series of questions to determine their pesticide exposure, including the type of pesticide, location of exposure (inside vs. outside the home), who applied the pesticide (self vs. a professional) and duration of pesticide use. Logistic regression models were used to estimate unadjusted and adjusted odds ratios (OR) and corresponding 95% confidence intervals (CI).

**Results:**

The most common pests encountered in participants' homes were ants, carpenter ants, and cockroaches. The calculated adjusted odds ratios for both self and professionally applied pesticides, specifically against the above mentioned insects, with regard to breast cancer risk were 1.25 (95% CI: 0.79-1.98) and 1.06 (95% CI: 0.65-1.73), respectively. Similarly, odds ratios and confidence intervals were calculated for other types of pesticides.

**Conclusions:**

Overall, the results of our study did not show an association between self-reported exposure to pesticides and breast cancer risk. Future studies, utilizing a larger sample size and more specific detail on time frame of pesticide exposure, are needed to further explore this question.

## Background

Self-reported exposure to pesticides and risk of cancer has been an area of much debate in epidemiological research. Recently a paper by Teitelbaum et al. [1] has again brought attention to this issue by showing a positive correlation between self-reported residential exposure to pesticides and risk of breast cancer. The possible role of organochlorine pesticides in the development of breast cancer has been widely studied over the past decades but no consistent evidence has been found to support this hypothesis [2]. In the northeastern United States, exposure to organochlorine compounds has been studied extensively as a possible cause for the higher rates of breast cancer observed in this geographic location [3-10]. These studies primarily focused on body burden of organochlorine pesticides, as measured in blood and adipose tissue. While earlier studies reported an association [11,12], numerous subsequent studies have revealed no convincing relationship [10,13]. Two studies in particular that were carried out on Long Island, New York, a region with somewhat elevated breast cancer rates, reported largely null associations between serum concentration of organochlorine pesticides and breast cancer. In a hospital-based study (American Health Foundation) conducted in the two largest hospitals on Long Island from 1994-1996, no relationship was observed between serum and adipose concentrations of organochlorine pesticides and development of breast cancer [4]. The Long Island Breast Cancer Study Project (LIBCSP), a subsequent large population-based case-control study of women living on Long Island conducted in 1996-1997, also did not reveal an increased risk of breast cancer in relation to serum organochlorine concentration [10].

Teitelbaum et al. [1] revisited the population based LIBCSP case-control study by conducting the first analysis of self-reported residential pesticide use and breast cancer risk. Although, the positive findings in this analysis seem to conflict with the generally null results from the biomarker studies of organochlorine pesticides and breast cancer risk, it may be valid for the following reasons. There are many types of commercial pesticides, which are available or have been available in the past, not all of which can be measured biochemically. Thus, the biomarker studies which were largely focused on organochlorine pesticides may not adequately assess exposure to other pesticides. In addition, there is a concern about the possibility of contaminants in these pesticides, which may have carcinogenic properties (e.g. polychlorinated dibenzo-p-dioxins (PCDD) in certain herbicides) [14]. Another possible explanation may be that there are inherent limitations of biomarkers in a case-control setting. The Institute of Medicine has cautioned that use of biomarker measures of body burden of organochlorine compounds such as dioxins and dibenzofurans long after initial exposure is likely to result in misclassification of exposure because of declines in tissue concentration over time, leading to a false-negative effect [15]. Furthermore, the majority of studies have examined the body burden of pesticides at the time of diagnosis (i.e. concomitant exposures rather than exposures closer to the time of disease initiation). Questionnaires may therefore have an advantage in exploring past exposures. Of course, the validity of self-reported exposures in epidemiology is always a concern, and there may have been a reporting bias among the LIBSCP patients diagnosed with breast cancer given the high profile and media coverage over the LIBSCP. Hence, we sought to analyze our own data on self-reported residential pesticide exposure using data from the American Health Foundation study to confirm the LIBSCP findings.

## Methods

### Study Participants

A case-control study was conducted at Long Island Jewish Medical Center and at North Shore University Hospital from October 1994 through October 1996. The results of this study have been described in detail previously [4]. These hospitals serve a patient population located mostly in New York City (primarily in the borough and county of Queens) as well as Nassau and Suffolk Counties. There were no restrictions on the residence status of the participants. All patients scheduled for breast biopsy or surgery were identified through frequent contacts with breast physicians at both participating hospitals and by consulting a list of patients scheduled for pre-surgical testing for operative procedures involving the breast. Cases were women with newly diagnosed breast cancer or carcinoma in-situ, while the controls included patients with benign breast disease or women undergoing non-breast related surgery. The study protocol was approved by the Institutional Review Boards of both hospitals and by the American Health Foundation. Patients were met at the pre-surgical units of both hospitals by trained interviewers who administered structured face-to-face interviews. More than 95% of the eligible patients agreed to participate. Questionnaires were completed for 447 cases (387 invasive breast cancer and 60 carcinoma in-situ) and 758 controls (490 benign breast disease and 268 surgical patients). The benign breast disease category included patients with benign breast neoplasm (n = 139), fibrocystic disease (n = 205), fibrosclerosis of the breast (n = 88), fibroadenosis of the breast (n = 10), solitary cyst of the breast (n = 7), and other non-neoplastic diseases of the breast. The 268 surgical control women were admitted for procedures involving the gallbladder (n = 106), removal of lipomas (n = 16), abdominal hernias (n = 22), osteoarthritis (n = 21) and other disorders unrelated to breast disease. Interviews were conducted before the surgical procedures were performed. Once the neoplastic diagnosis was confirmed, based on pathology reports, study subjects were classified as either case or control patients. This classification strategy was utilized in order to decrease the possibility of bias since the interviewer, patient, investigator, and physician were unaware of the disease diagnosis at the time of interview.

### Data Collection

Trained interviewers used a structured questionnaire to collect data which included known risk factors for breast cancer such as age at diagnosis, family history of cancer in first-degree relatives, body mass index, menstrual history and oral contraceptive use. Though family history of all types of cancer was taken, only family history of breast cancer was included in the final statistical analysis.

All patients who reported pesticide use were then asked a series of questions on pesticide exposure, which included the types of pesticides to which the patient was exposed, whether it was applied by a professional insect exterminator or the patient herself, delivery method, and location of dispersal. Types of pesticides, including both insecticides and herbicides, were classified into seven categories. These included agents used for:

1. Ants, carpenter ants, cockroaches

2. Bees or wasps

3. Flies or mosquitoes in the home or yard

4. Fleas or ticks in the home

5. Weeds

6. Lawn insects

7. Insects or diseases of outdoor plants

If the patient reported the use of any of the above pesticides in the past, then specific questions were asked regarding the period of exposure. These included, how many months of the year pesticides were used and how often they were used during those months, with possible responses ranging from daily or continuous use to less than once-per-week. It was uncertain whether the risk of breast cancer would be greater with a professional application since higher concentrations of pesticides are often used, or with off-the-shelf agents since the patient would be performing the pesticide application herself. Likewise, data was collected regarding various product types since particle density and pesticide dose could not be assumed to be uniform across different application modalities. It should be noted that our analysis was limited by the data collection tool not specifically addressing the start and stop years of pesticide use. Thus, it was not possible to know the timing of first exposure and to estimate the lifetime exposure.

### Statistical Analysis

For each of the seven pest categories, we calculated the risk of breast cancer according to who applied the pesticide, the method of application, and the location of application (inside or outside the home). The frequency of exposure was estimated by asking the subject how many months of the year pesticides were used and during those months how often the product was used. The answers to both these questions were used to generate an estimate of cumulative exposure. This calculated exposure density was arranged in progressive tertiles, and then analyzed for association to breast cancer risk.

Furthermore, some patients may have been exposed to multiple products. Thus, exposure categories could not be mutually exclusive. The most common mode of application was by sprayer. The use of foggers and powders was infrequent; therefore data from these two groups was combined into a single categorical variable. Similarly, few subjects reported applying pesticides both inside and outside the home. So, these subjects were combined with participants who reported using pesticides only inside the house. Data as to who actually applied the pesticide was divided into application of pesticides by the study participant and application by other individuals, who may have been pest-control professionals or household members other than the study participant. In pesticide-specific analysis, persons who reported not being exposed to the specific pesticide under analysis were classified as the non-exposed referent group.

Logistic regression models were used to estimate adjusted and unadjusted odds ratios (OR) and corresponding 95% confidence intervals (CI), regarding associations between pesticide use and development of breast cancer. Specifically, unconditional logistic regression models were selected to determine the risk according to 1) who applied the product, 2) what type of product was applied, or 3) where the product was applied. The models were adjusted for the following confounding variables: subject's age at the time of disease diagnosis, race, religion, level of education, family history of breast cancer, body mass index, age at menopause, and age at menarche. A separate model was created to test the effect of cumulative exposure in comparison to those who were never exposed. For each of the seven categories of pests, cumulative exposure to pesticide was calculated and then categorized into upper, middle and lower tertiles.

Finally, a third model was developed based on multiple exposures (yes/no) to all pesticides. For this analysis, subjects were assigned to one of the following categories: never exposed to pesticides, exposed to one type of pesticide, exposed to two types of pesticides, exposed to three types of pesticides, exposed to four types of pesticides, or exposed to five or more types of pesticides. The never-exposed category only included subjects who reported not being exposed to any pesticide. A test for trend was conducted across these categories using the Cochran-Armitage test. All data analyses were conducted using SAS statistical software version 9.1 (Cary, NC).

## Results

A total of 1205 patients were included in this study. Regarding the age of study subject 30% of the cases and 48% of the controls were younger than 50 years of age, respectively and 20% of cases and 12% of controls were older than age of 70, respectively. As regard to menopause, 42% of cases and 51% of controls had menopause by 49 years of age, respectively. Level of education was used as a substitute for the socioeconomic status. There were no significant differences between cases and controls with regard to religious status, race, oral contraceptive use, or age at menarche (Table 1).

**Table 1:** Characteristics of the study population

Variables		Cases (%)	Controls (%)
Age	< 50	136 (30.4)	361 (47.6)
	
	50-59	121 (27.1)	173 (22.8)
	
	60-69	101 (22.6)	132 (17.4)
	
	> 70	89 (19.9)	92 (12.1)

Education	≤High school	175 (41.6)	261 (34.8)
	
	≤College graduate	157 (37.3)	321 (42.9)
	
	Post graduate	89 (21.14)	167 (22.3)

Race	White	384 (91.2)	689 (92.0)
	
	Black	29 (6.9)	56 (7.5)
	
	Asian/Others	8 (1.9)	4 (0.5)

Religion	Protestant	77 (18.3)	134 (17.9)
	
	Catholics	167 (39.7)	330 (44.1)
	
	Jewish	146 (34.7)	231 (30.8)
	
	Others	25 (5.9)	39 (5.2)
	
	None	4 (1.0)	12 (1.6)
	
	Refused	2 (0.5)	3 (0.4)

BMI*	< 25	200 (48.19)	370 (50.3)
	
	25-29.9	124 (29.9)	206 (28.0)
	
	≥30	91 (21.9)	160 (21.7)

Menstrual status	Pre menopausal	114 (27.1)	307 (41.0)
	
	Peri menopausal	41 (9.7)	70 (9.4)
	
	Postmenopausal	266 (63.2)	372 (49.7)

Age at menopause	≤ 49	110 (42.3)	188 (51.0)
	
	50	45 (17.3)	57 (15.4)
	
	≥51	105 (40.4)	124 (33.6)

Age at menarche	< 12	216 (51.3)	373 (49.9)
	
	≥12	205 (48.7)	375 (50.1)

Ever used an Oral Contraceptive	Yes	193 (45.8)	369 (49.5)
	
	No	228 (54.2)	376 (50.5)

*BMI = body mass index.

The total numbers of subjects never exposed to any kind of pesticide were 143 cases (32%) and 229 controls (30%), respectively. When the relationship between breast cancer and the type of pesticides used was analyzed with regard to who applied the product, both the unadjusted and adjusted odds ratios were not found to be significant. For example, the most commonly used pesticides were for ant, carpenter ant, and cockroach infestations. The adjusted odds ratio for self-application of pesticide and application by other individuals were 1.25 (95% CI: 0.79-1.98) and 1.06 (95% CI: 0.65-1.73), respectively (Table 2). Similarly, when different pesticide application methods were analyzed with regard to breast cancer risk, no statistically significant association was found. For example, the breast cancer risk associated with application by spray in the ants and cockroaches category was 1.27 (95% CI: 0.83-1.94); and by fogger or powder together in the same category was 0.81 (95% CI: 0.44-1.49) (Table 3). The breast cancer risk for pesticides against ant, carpenter ant, and cockroach infestations, when pesticide application occurred either inside the home or both inside and outside the home was 1.13 (95% CI: 0.75-1.72), while the breast cancer risk for outside-only application of pesticide was 1.22 (95% CI: 0.60-2.50) (Table 4).

**Table 2:** Exposure to pesticides based on who applied the product

Types of pesticides used for the following pests	Who applied these products	Number of cases	Number of control	Adjusted OR*	95% CI
Never exposed^+^	__	143	229	__	__

Ants, carpenter ant, cockroaches	Study participant	120	205	1.25	0.79-1.98
	
	Another from the same household/professional	109	108	1.06	0.65-1.73

Bees or wasps	Study participant	22	41	0.73	0.31-1.72
	
	Another from the same household/professional	27	42	1.66	0.70-3.94

Flies or mosquitoes in your home or yard	Study participant	19	28	2.01	0.71-5.66
	
	Another from the same household/professional	13	12	1.66	0.42-6.62

Fleas or ticks in your home	Study participant	27	41	1.52	0.58-3.93
	
	Another from the same household/professional	15	36	1.59	0.57-4.44

Weeds	Study participant	12	18	1.2	0.40-3.58
	
	Another from the same household/professional	104	164	1.21	0.75-1.97

Lawn insects	Study participant	11	7	2.68	0.72-9.92
	
	Another from the same household/professional	89	133	1.22	0.73-2.04

Insects or diseases of outdoor plants	Study participant	18	17	2.55	0.81-8.00
	
	Another from the same household/professional	57	99	0.95	0.52-1.74

* Adjusted for age at the time of diagnosis, race, religion, level of education, family history of breast cancer, body mass index, age at menopause and age at menarche.

^+ ^The never exposed category included persons who reported not being exposed to the pesticides.

CI, confidence interval; OR, odds ratio

**Table 3:** Exposure to pesticides analyzed based on the type of product used

Types of pesticides used for the following pests	Type of product	Number of cases	Number of control	Adjusted OR	95% CI
Never exposed^+^	__	143	229	__	__

Ants, carpenter ant, cockroaches	spray	181	296	1.27	0.83-1.94
	
	fogger/powder/some other form	46	88	0.81	0.44-1.49

Bees or wasps	spray	49	78	1.11	0.58-2.13
	
	fogger/powder/some other form	0	5	0	__

Flies or mosquitoes in your home or yard	spray	29	33	2.16	0.88-5.30
	
	fogger/powder/some other form	2	7	< 0.001	__

Fleas or ticks in your home	spray	17	30	1.85	0.60-5.68
	
	fogger/powder/some other form	25	46	1.4	0.57-3.43

Weeds	spray	71	123	0.94	0.55-1.61
	
	fogger/powder/some other form	43	58	1.76	0.91-3.42

Lawn insects	spray	80	113	1.2	0.70-2.03
	
	fogger/powder/some other form	20	26	1.99	0.81-4.86

Insects or diseases of outdoor plants	spray	67	103	1.03	0.57-1.85
	
	fogger/powder/some other form	8	13	2.55	0.60-10.88

* Adjusted for age at the time of diagnosis, race, religion, level of education, family history of breast cancer, body mass index, age at menopause and age at menarche.

^+ ^The never exposed category included persons who reported not being exposed to the pesticides.

CI, confidence interval; OR, odds ratio

**Table 4:** Exposure to pesticides based on where it was applied

Types of pesticides used for the following pests	Location	Number of cases	Number of control	Adjusted OR	95% CI
Never exposed^+^	__	143	229	__	__

Ants, carpenter ant, cockroaches	Inside or both inside and outside	200	343	1.13	0.75-1.72
	
	Outside only	28	42	1.22	0.60-2.50

Bees or wasps	Inside or both inside and outside	15	21	2.42	0.76-7.75
	
	Outside only	34	61	0.84	0.40-1.74

Flies or mosquitoes in your home or yard	Inside or both inside and outside	17	17	2.21	0.77-6.30
	
	Outside only	13	23	1.15	0.29-4.60

Fleas or ticks in your home	Inside or both inside and outside	40	70	1.55	0.71-3.40
	
	Outside only	2	7	1.57	0.21-11.94

Weeds	Inside or both inside and outside	3	2	4.77	0.37-62.10
	
	Outside only	113	177	1.21	0.76-1.93

Lawn insects	Inside or both inside and outside	3	1	__	__
	
	Outside only	97	138	1.3	0.79-2.14

Insects or diseases of outdoor plants	Inside or both inside and outside	5	3	__	__
	
	Outside only	69	112	1.05	0.59-1.85

* Adjusted for age at the time of diagnosis, race, religion, level of education, family history of breast cancer, body mass index, age at menopause and age at menarche.

^+ ^The never exposed category included persons who reported not being exposed to the pesticides.

CI, confidence interval; OR, odds ratio

With regard to the relationship between cumulative exposure to a single pesticide and risk of breast cancer, no significant association was found (Table 5). Furthermore, no dose-response relationship was discerned for any type of pesticide employed. For example, for ants, carpenter ants and cockroaches; the risk of breast cancer was 0.99 (95% CI: 0.56-1.72) in tertile one, 1.15 (95% CI: 0.56-2.36) in tertile two, and 0.95 (95% CI: 0.52-1.73) in tertile three. With regard to exposure to multiple types of pesticide, similar to single pesticide exposure, no dose-response relationship could be found. Likewise, the trend test for a possible association did not show a significant relationship (unadjusted p-value was 0.28; adjusted p-value was 0.31) (Table 6).

**Table 5:** Cumulative exposure to different types of pesticides

Types of pesticides used for the following pests	Cumulative Exposures	Number of cases	Number of controls			OR after adjusting for confounders* (vs. never exposed)	95% CI
					
			Benign breast diseases	Other surgical diseases	Total		
Never exposed^+^	__	143	145	84	229	__	__

Ants, carpenter ants, cockroaches	Tertiles 1 (1-33%)	93	105	39	144	0.99	0.56-1.72
	
	Tertiles 2 (34-66%)	49	51	37	88	1.15	0.56-2.36
	
	Tertiles 3 (67-100%)	84	97	54	151	0.95	0.52-1.73

Bees or wasps	Tertiles 1 (1-33%)	25	28	11	39	0.7	0.28-1.75
	
	Tertiles 2 (34-66%)	5	14	9	23	0.45	0.11-1.81
	
	Tertiles 3 (67-100%)	19	10	10	20	3.15	0.61-16.35

Flies or mosquitoes in your home or yard	Tertiles 1 (1-33%)	14	7	5	12	2.37	0.50-11.22
	
	Tertiles 2 (34-66%)	8	6	6	12	1.18	0.20-6.98
	
	Tertiles 3 (67-100%)	9	9	7	16	1.66	0.26-10.67

Fleas or ticks in your home	Tertiles 1 (1-33%)	17	18	13	31	1.1	0.30-3.95
	
	Tertiles 2 (34-66%)	15	8	9	17	10.73	1.14-101.28
	
	Tertiles 3 (67-100%)	10	19	7	26	2.95	0.46-19.04

Weeds	Tertiles 1 (1-33%)	51	49	34	83	1.21	0.61-2.40
	
	Tertiles 2 (34-66%)	22	24	13	37	1.7	0.64-4.53
	
	Tertiles 3 (67-100%)	41	37	21	58	1.03	0.43-2.45

Lawn insects	Tertiles 1 (1-33%)	42	41	19	60	1.43	0.67-3.05
	
	Tertiles 2 (34-66%)	20	16	12	28	2.42	0.86-6.78
	
	Tertiles 3 (67-100%)	36	30	20	50	0.68	0.28-1.64

Insects or diseases of outdoor plants	Tertiles 1 (1-33%)	26	37	21	58	0.68	0.29-1.58
	
	Tertiles 2 (34-66%)	14	12	6	18	1.13	0.37-3.40
	
	Tertiles 3 (67-100%)	33	22	18	40	1.04	0.37-2.92

* Adjusted for age at the time of diagnosis, race, religion, level of education, family history of breast cancer, body mass index, age at menopause and age at menarche.

^+ ^The never exposed category included persons who reported not being exposed to the pesticides.

CI, confidence interval; OR, odds ratio

**Table 6:** Exposure to one or more different types of pesticides

Number of pesticides exposed to	Cases n = 446 (%)	Control n = 758 (%)	Adjusted OR*	95% CI
No exposure to any type of pesticide (never said yes)	143 (32.0)	229 (30.2)	__	__

Exposure to one type of pesticide only	141 (31.5)	285 (37.6)	0.98	0.64-1.52

Exposure to two different types of pesticides	64 (14.3)	96 (12.7)	1.45	0.80-2.61

Exposure to three different types of pesticides	50 (11.2)	78 (10.3)	1.25	0.70-2.25

Exposure to four different types of pesticides	32 (7.2)	52 (6.9)	0.75	0.36-1.57

Exposure to five to seven different types of pesticides	17 (3.8)	18 (2.4)	2.21	0.80-6.09

				

**TREND TEST**	p-value^+ ^0.31			

* Adjusted for age at the time of diagnosis, race, religion, level of education, family history of breast cancer, body mass index, age at menopause and age at menarche.

^+^significant p value defined as < 0.05

CI, confidence interval; OR, odds ratio

Finally, separate analyses were performed for breast cancer risk with respect to control patients who either had benign breast diseases only or control patients who had been diagnosed with non-breast related surgical conditions. The findings were consistent with those found when both control groups were combined to form a single control group.

## Discussion

In our hospital-based case-control study, no significant relationship was found despite performing an analysis which took into consideration the location of exposure, method of pesticide application, and who applied the pesticide or herbicide. However, an increased risk of breast cancer was found in those women who had a moderate level of exposure to pesticides that were used for flea and tick infestations (odds ratio 10.73 (95% CI: 1.14-101.28) (Table 5). This finding is likely due to chance since it does not follow a clear dose-response relationship and is not consistent with our other findings.

Organochlorine pesticides have received more attention in the past because of their persistence in the environment, continued detection in the food supply and breast milk and their ability to be stored in the adipose tissue of both animals and humans [16]. Furthermore, because some organochlorine compounds have been shown to act as both estrogen agonists or antagonists in several animal experiments, a possible association of breast cancer risk with organochlorine exposure has been hypothesized and investigated [17,18]. The collective evidence to date does not appear to support a relation between exposure to organochlorines and risk of breast cancer but several questions still remain regarding the time frame of pesticide exposure [16,19-21]. The shorter lived compounds, unlike the organochlorines, do not persist in the environment for long durations. However, these shorter lived pesticides may still be hazardous with longstanding biological effects, and self-reporting is often the only means of assessing historical exposure to such chemical in epidemiological studies [22].

Comparison between our study and the LIBSCP study [1] shows conflicting results. The LIBSCP study showed an increased breast cancer risk with all pest groups combined, (OR = 1.39, 95% CI: 1.15-1.68), but there was no evidence of increasing risk with increasing lifetime exposure. However, this weak association was not supported by our study, which was also performed in the Long Island, New York area. In comparison to our study, Teitelbaum's study had a larger sample size and employed a measure of lifetime exposure, which enumerated the number of years for which the pesticide was applied. In our study, the estimated cumulative exposure represented the frequency of pesticide exposure in a given year. Unlike Teitelbaum's study, our questionnaire did not address how many years each product had been used, thus it was not possible to estimate lifetime exposure. Similar to the Teitelbaum's study, our study showed that the risk varied little between different types of products or who applied the agent.

One possible explanation for the difference in findings between our study and the LIBCSP study is that LIBSCP used randomly selected population controls. In our study, women with a suspicious breast mass did not know their tissue diagnosis at the time of questionnaire completion. Thus, the design of our study may have minimized recall bias. Furthermore, positive findings in the LIBCSP study could have been due to chance as the authors note that they did not observe a dose-response relationship.

The major strength of our study is that the data was collected in-person, by an interviewer before the disease diagnosis was established and assignment to either the case or control group occurred after the review of tissue pathology reports. This potentially would have minimized any possibility of a reporting bias, despite the high profile of breast cancer risk studies from Long Island, NY. The use of two control groups serves to validate these findings, where subjects with benign breast disease likely had very similar referral patterns as breast cancer cases, and the use of general surgical controls reduced possible methodological bias if an association between pesticide use and benign breast disease existed.

The main limitation of our study is that the questionnaire did not specify the time frame of exposure. This might be important since we know that there is potential of a long lag time between exposure to pesticides and development of breast cancer, as reported by Hoyer et al. [12]. This lack of specificity does limit application of our results. In addition, we did not have information regarding the chemicals used in each of our seven categories of pesticides, limiting the mutual exclusivity of categories. With respect to sample size, the small sample size of our study population might have limited our ability to discern small exposure effects. Also, separating the pesticides into smaller categories might have reduced the statistical power even further. However, the general pattern of odds ratios across the many categories is largely null indicating an overall lack of association. Finally, self-reporting is a crude measure of historical environmental exposure and not of an actual biological exposure, although the validity of biological measurements may also be somewhat problematic due to degradation and elimination of compounds over time.

## Conclusions

In conclusion, our case-control study did not show an association between self-reported residential exposure to pesticides and breast cancer risk. The real strength of this study is that the interviews were conducted before tissue-based diagnosis of disease. However, our study was limited by the lack of detail on the time of initial exposure, a smaller sample size, and an absence of information regarding the chemical constituents of different categories of pesticides. Therefore, we recommend that a follow-up study with more precise information about the time frame of exposure, a larger sample size, and a detailed account of chemical constituents of the pesticides should be conducted to further explore this question.

## Abbreviations

LIBCSP: Long Island Breast Cancer Study Project.

## Competing interests

The authors declare that they have no competing interests.

## Authors' contributions

UF participated in study design, prepared the datasets, oversaw the analysis and drafted the manuscript. MJ participated in study design, and helped drafting the manuscript. VN participated in study design, and helped drafting the manuscript. PC participated in the study design, prepared the datasets, oversaw the analysis and, helped drafting the manuscript. DF reviewed the manuscript and revised it critically for important intellectual content. NJG helped prepare the datasets and performed the statistical analysis. SDS was principal investigator, reviewed the manuscript and revised it critically for important intellectual content. JM participated in the study design, oversaw the analysis, reviewed the study results, and revised the manuscript critically for important intellectual content.

All authors read and approved the final manuscript.

## References

[B1] Teitelbaum SL Gammon MD Britton JA Neugut AI Levin B Stellman SD Reported residential pesticide use and breast cancer risk on Long Island, New York Am J Epidemiol 2007 165 643 651 10.1093/aje/kwk046 17166928

[B2] Salehi F Turner MC Phillips KP Wigle DT Krewski D Aronson KJ Review of the etiology of breast cancer with special attention to organochlorines as potential endocrine disruptors J Toxicol Environ Health B Crit Rev 2008 11 276 300 18368557 10.1080/10937400701875923

[B3] Zheng T Holford TR Mayne ST Ward B Carter D Owens PH Dubrow R Zahm SH Boyle P Archibeque S DDE and DDT in breast adipose tissue and risk of female breast cancer Am J Epidemiol 1999 150 453 458 10472944 10.1093/oxfordjournals.aje.a010033

[B4] Stellman SD Djordjevic MV Britton JA Muscat JE Citron ML Kemeny M Busch E Gong L Breast cancer risk in relation to adipose concentrations of organochlorine pesticides and polychlorinated biphenyls in Long Island, New York Cancer Epidemiol Biomarkers Prev 2000 9 1241 1249 11097233

[B5] Zheng T Holford TR Tessari J Mayne ST Owens PH Ward B Carter D Boyle P Dubrow R Archibeque-Engle S Zahm SH Breast Cancer Risk Associated with Congeners of Polychlorinated Biphenyls Am J Epidemiol 2000 152 50 58 10.1093/aje/152.1.50 10901329

[B6] Wolff MS Berkowitz GS Brower S Senie R Bleiweiss IJ Tartter P Pace B Roy N Wallenstein S Weston A Organochlorine exposures and breast cancer risk in New York City women Environ Res 2000 84 151 161 10.1006/enrs.2000.4075 11068929

[B7] Moysich KB Ambrosone CB Vena JE Shields PG Mendola P Kostyniak P Greizerstein H Graham S Marshall JR Schisterman EF Freudenheim JL Environmental organochlorine exposure and postmenopausal breast cancer risk Cancer Epidemiol Biomarkers Prev 1998 7 181 188 9521429

[B8] Helzlsouer KJ Alberg AJ Huang HY Hoffman SC Strickland PT Brock JW Burse VW Needham LL Bell DA Lavigne JA Yager JD Comstock GW Serum concentrations of organochlorine compounds and the subsequent development of breast cancer Cancer Epidemiol Biomarkers Prev 1999 8 525 532 10385143

[B9] Wolff MS Zeleniuch-Jacquotte A Dubin N Toniolo P Risk of breast cancer and organochlorine exposure Cancer Epidemiol Biomarkers Prev 2000 9 271 277 10750665

[B10] Gammon MD Wolff MS Neugut AI Eng SM Teitelbaum SL Britton JA Terry MB Levin B Stellman SD Kabat GC Hatch M Senie R Berkowitz G Bradlow HL Garbowski G Maffeo C Montalvan P Kemeny M Citron M Schnabel F Schuss A Hajdu S Vinceguerra V Niguidula N Ireland K Santella RM Environmental toxins and breast cancer on Long Island. II. Organochlorine compound levels in blood Cancer Epidemiol Biomarkers Prev 2002 11 686 697 12163320

[B11] Hoyer AP Jorgensen T Grandjean P Hartvig HB Repeated measurements of organochlorine exposure and breast cancer risk (Denmark) Cancer Causes Control 2000 11 177 184 10.1023/A:1008926219539 10710203

[B12] Hoyer AP Grandjean P Jorgensen T Brock JW Hartvig HB Organochlorine exposure and risk of breast cancer Lancet 1998 352 1816 1820 10.1016/S0140-6736(98)04504-8 9851382

[B13] Calle EE Frumkin H Henley SJ Savitz DA Thun MJ Organochlorines and breast cancer risk CA Cancer J Clin 2002 52 301 309 10.3322/canjclin.52.5.301 12363327

[B14] Dich J Zahm SH Hanberg A Adami HO Pesticides and cancer Cancer Causes Control 1997 8 420 443 10.1023/A:1018413522959 9498903

[B15] Institute of Medicine, Committee to Review the Health Effects in Vietnam Veterans of Exposure to Herbicides. Veterans and Agent Orange: Health Effects of Herbicides Used in Vietnam 1994 National Academy of Sciences Press, Washington, D.C. 283

[B16] Snedeker SM Pesticides and breast cancer risk: a review of DDT, DDE, and dieldrin Environ Health Perspect 2001 109 Suppl 1 35 47 10.2307/3434845 11250804 PMC1240541

[B17] Brody JG Aschengrau A McKelvey W Rudel RA Swartz CH Kennedy T Breast cancer risk and historical exposure to pesticides from wide-area applications assessed with GIS Environ Health Perspect 2004 112 889 897 15175178 10.1289/ehp.6845 PMC1242018

[B18] Davis DL Bradlow HL Wolff M Woodruff T Hoel DG Anton-Culver H Medical hypothesis: xenoestrogens as preventable causes of breast cancer Environ Health Perspect 1993 101 372 377 10.2307/3431889 8119245 PMC1519851

[B19] Eskenazi B Chevrier J Rosas LG Anderson HA Bornman MS Bouwman H Chen A Cohn BA de Jager C Henshel DS Leipzig F Leipzig JS Lorenz EC Snedeker SM Stapleton D The Pine River statement: human health consequences of DDT use Environ Health Perspect 2009 117 1359 1367 19750098 10.1289/ehp.11748 PMC2737010

[B20] Engel LS Hill DA Hoppin JA Lubin JH Lynch CF Pierce J Samanic C Sandler DP Blair A Alavanja MC Pesticide use and breast cancer risk among farmers' wives in the agricultural health study Am J Epidemiol 2005 161 121 135 10.1093/aje/kwi022 15632262

[B21] Cohn BA Wolff MS Cirillo PM Sholtz RI DDT and breast cancer in young women: new data on the significance of age at exposure Environ Health Perspect 2007 115 1406 1414 17938728 10.1289/ehp.10260 PMC2022666

[B22] Engel LS Seixas NS Keifer MC Longstreth WT Jr Checkoway H Validity study of self-reported pesticide exposure among orchardists J Expo Anal Environ Epidemiol 2001 11 359 368 10.1038/sj.jea.7500176 11687909

